# Research on Augmentation of Wood Microscopic Image Dataset Based on Generative Adversarial Networks

**DOI:** 10.3390/jimaging11120445

**Published:** 2025-12-12

**Authors:** Shuo Xu, Hang Su, Lei Zhao

**Affiliations:** College of Science and Information, Qingdao Agricultural University, Qingdao 266109, China; xushuo@stu.qau.edu.cn (S.X.); sh10913@163.com (H.S.)

**Keywords:** deep learning, neural networks, generative adversarial networks, microscopic images, data augmentation

## Abstract

Microscopic wood images are vital in wood analysis and classification research. However, the high cost of acquiring microscopic images and the limitations of experimental conditions have led to a severe problem of insufficient sample data, which significantly restricts the training performance and generalization ability of deep learning models. This study first used basic image processing techniques to perform preliminary augmentation of the original dataset. The augmented data were then input into five GAN models, BGAN, DCGAN, WGAN-GP, LSGAN, and StyleGAN2, for training. The quality and model performance of the generated images were assessed by analyzing the degree of fidelity of cellular structure (e.g., earlywood, latewood, and wood rays), image clarity, and diversity of the images for each model-generated image, as well as by using KID, IS, and SSIM. The results showed that images generated by BGAN and WGAN-GP exhibited high quality, with lower KID values and higher IS values, and the generated images were visually close to real images. In contrast, the DCGAN, LSGAN, and StyleGAN2 models experienced mode collapse during training, resulting in lower image clarity and diversity compared to the other models. Through a comparative analysis of different GAN models, this study demonstrates the feasibility and effectiveness of Generative Adversarial Networks in the domain of small-sample image data augmentation, providing an important reference for further research in the field of wood identification.

## 1. Introduction

With the rapid increase in global timber trade, the trade of timber species regulated by the Convention on International Trade in Endangered Species of Wild Fauna and Flora (CITES) has become a global focus. In 2023, the total global trade in timber and paper products reached 482 billion USD, with industrial roundwood trade totaling 100 million cubic meters and sawnwood trade reaching 129 million cubic meters [1]. As the gatekeepers of international trade, customs play a crucial role in regulating timber imports and exports, preventing illegal logging, and ensuring the enforcement of CITES. Currently, the mismatch of wood species has become one of the most common forms of fraud in the timber trade [2]. Some timber importers, lacking professional knowledge, may purchase inferior timber at high prices, leading to significant financial losses. Customs authorities worldwide also need to identify timber species to combat illegal logging and related trade, ensuring better enforcement of CITES. Therefore, there is a need to establish efficient timber identification methods for the rapid and accurate identification of timber species.

Traditional timber identification methods mainly rely on experienced professionals who observe the wood’s cellular structure through the naked eye or a microscope, identifying characteristics such as vessel density, diameter, and wood ray width [3]. However, this method is time-consuming, labor-intensive, and requires high expertise, often leading to misidentifications, making it challenging to meet the demand for large-scale timber classification [4]. Computer vision, a technology that uses devices such as cameras and image sensors to capture image information and analyzes and processes this data through algorithms [5,6], can be applied in timber identification. In this field, computer vision technology can capture features such as texture and color on the wood surface, automatically extracting and classifying these features through algorithms. Deep learning, a major branch of computer vision, can achieve high-precision timber classification and recognition by using deep learning models such as Convolutional Neural Networks (CNNs) to learn complex features from timber images automatically [7]. For example, Sun et al. [8] proposed a method using LDA for dimensionality reduction of image features extracted by a pre-trained CNN, followed by classification using machine learning classifiers; Wu et al. [9] applied deep learning architectures such as DenseNet, achieving an accuracy rate of 98.2% in hardwood classification. Pan et al. [10] combined multi-scale CNNs and portable near-infrared spectrometers (NIR) to propose a non-destructive, rapid timber identification method, which successfully enhanced feature extraction capability and achieved a classification accuracy of 97.34%. Similar studies, including those by Ravindran et al. [2], developed deployable recognition systems that efficiently classify CITES-protected timber species, providing practical solutions to combat illegal logging. Integrating deep learning with image processing technologies significantly improves recognition accuracy and enables rapid response in field deployments, serving as crucial technological support for timber protection and trade regulation.

Microscopic wood slices have long been used for forensic timber identification, analyzing important timber characteristics such as permeability and processability with chemical agents and understanding the timber’s functionality [11]. Using microscopic images for recognition allows for precise representation of microstructural features, improving the accuracy of timber species differentiation, particularly for morphologically similar or processed timber samples. However, only a few datasets are publicly available for research and development. One of the most widely used benchmark datasets for timber identification is the one created by Martins et al. [12]. The acquisition of microscopic images is costly and subject to limitations such as noise, lighting variations, and specific equipment constraints [13], resulting in limited quality and diversity of datasets for microscopic wood images.

In deep learning-based timber recognition, large-scale datasets are essential to ensure good generalization and accuracy of the model. Therefore, the quality and quantity of data in the dataset are critical to the success of model training. To overcome the limitations posed by dataset quality and data quantity in classification models, researchers have begun exploring methods for dataset augmentation and enhancement. Generative Adversarial Networks (GANs), proposed by Goodfellow et al. [14] in 2014, is a special type of deep learning model that generates highly realistic and diverse images by alternately training two adversarial neural networks. GANs have been widely applied in various fields, including medical analysis [15], underwater scene analysis [16], face recognition [17], food safety [18], and agriculture [19].

In recent years, GANs have made breakthroughs in the fields of image generation and transformation, showing remarkable capabilities in diverse applications. Classical GAN models generate realistic, high-quality images through adversarial learning, but they also face challenges such as unstable training and mode collapse [20]. To address these issues, researchers have proposed various improvements. For example, He et al. [21] introduced IntroGAN, which combines incremental learning and prototype classification to achieve mutual enhancement in both generation and classification, while Fathallah et al. [20] improved GAN training stability and performance by introducing identity blocks and optimized loss functions. The training quality of GANs in limited data scenarios has also received significant attention. For instance, Israr et al. [22] proposed a self-supervised consistency regularization-based approach to achieve high-fidelity transfer of diversity from the source domain to the target domain. Similarly, Yildiz et al. [23] developed a single-image GAN model combining self-attention mechanisms and DenseNet architectures, demonstrating its ability to generate high-quality images under limited data. Additionally, GANs have yielded fruitful results in specific applications. For example, Sun et al. [24] used an improved CycleGAN to effectively suppress random seismic noise, enhancing feature extraction capability through a fusion of ResNet and Unet architectures. Chen et al. [25] proposed the CA-GAN, which combines content space and attribute space decoupling mechanisms to generate Chinese traditional art paintings, further improving image resolution and artistic quality. With its powerful ability to generate highly realistic and diverse images, GAN has demonstrated incomparable advantages across various fields. However, there has been relatively limited application of GANs in the timber field, especially for microscopic images at the cellular level, which restricts further improvement in the accuracy of timber species recognition.

While the comparative performance of these GAN variants is increasingly well-understood for natural images, their behavior in highly specialized, data-scarce scientific domains remains largely uncharted. This gap is particularly critical in wood microscopy, where the stochastic textures of cellular structures (e.g., earlywood, latewood, wood rays) and the extreme difficulty of acquiring large-scale datasets present unique challenges that are not addressed by general-purpose image generation studies. Simply transferring findings from other domains is inadequate; a dedicated, systematic investigation is required to establish a reliable foundation for applying generative models to wood science. Therefore, this work presents the first comprehensive, cross-architecture benchmark specifically for microscopic wood imagery, executed under the challenging scenario of extreme data scarcity.

In this work, to address the limitations of dataset quality and data quantity on classification models, particularly the challenges in constructing wood microscopic image datasets, we propose the use of GANs for dataset augmentation and enhancement. Several GAN models, including boundary-seeking GAN (BGAN) [26], Deep Convolutional GAN (DCGAN) [27], Wasserstein GAN with Gradient Penalty (WGAN-GP) [28], Least Squares GAN (LSGAN) [29], and StyleGAN2 [30], are trained and used to solve the problem of limited dataset quality and data quantity in the current timber species recognition process. A comparative summary of these models, highlighting their core contributions and main limitations as identified in the literature, is provided in Table 1. The study analyzes the realism of cell structures (such as earlywood, latewood, and wood rays) generated by each model, along with image clarity, diversity, and quality. The image quality and model performance are assessed using metrics such as Kernel Inception Distance (KID), Inception Score (IS), and Structural Similarity Index Measure (SSIM), enabling a comparison to determine the most effective model for augmenting the wood microstructure image dataset.

The unique contributions of this work can be summarized as follows:

(1) We provide the first systematic, cross-architecture evaluation of multiple GAN models for microscopic wood imagery under extreme limited-data conditions, with a specific focus on softwood species. This distinguishes our work from prior studies like Lopes et al. [11], which focused on hardwood species, and addresses the distinct anatomical characteristics and associated generation challenges of softwoods.
(2) We introduce a domain-adapted evaluation framework that incorporates woody anatomical characteristics via KID using DenseNet wood embeddings, LPIPS diversity, and Density–Coverage metrics—evaluation dimensions not addressed in Lopes et al. [11].
(3) We analyze model robustness, distributional coverage, and failure modes specific to microscopic textures, offering practical insights for real-world small-sample wood identification augmentation.

## 2. Materials and Methods

### 2.1. Wood Microscopic Image Dataset

In this study, we used a publicly accessible wood microscopic image dataset, which is available from the Federal University of Paraná (UFPR) database in Brazil. The dataset includes both macro images and microscopic wood images. It was created as a benchmark for automated timber identification research and can be accessed from the website of the Federal University of Paraná [12].

**Figure 1:**
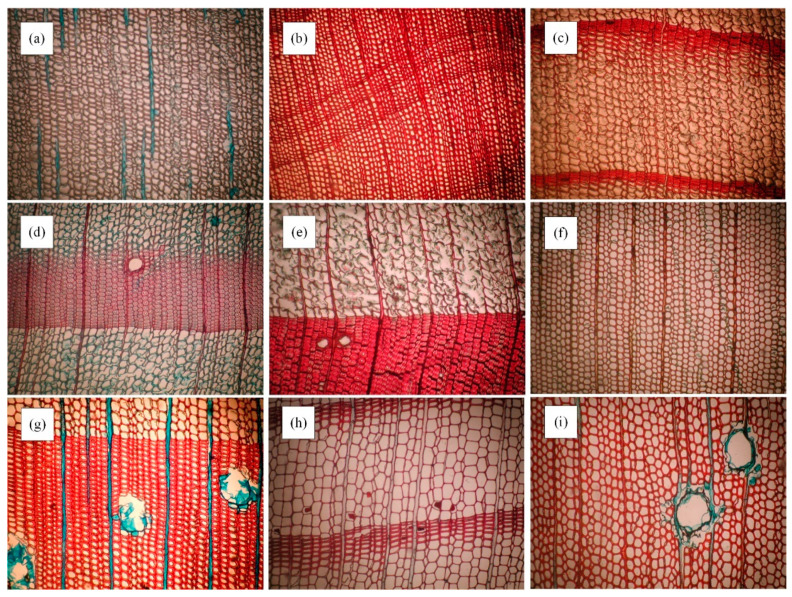
Sample Microscopic Images from the UFPR Wood Dataset. (**a**) Ginkgo biloba; (**b**) Cephalotaxus drupacea; (**c**) Chamaecyparis formosensis; (**d**) Larix lariciana; (**e**) Larix leptolepis; (**f**) Cupressus lindleyi; (**g**) Pinus elliottii; (**h**) Sequoia sempervirens; (**i**) Pinus caribaea. Refer to the dataset for full dataset description.

Figure 1 shows microscopic images of wood samples from nine species in the dataset. The images display features such as the size and arrangement of vessels, axial parenchyma cells, and rays—key characteristics for classifying microscopic wood images. The UFPR database consists of 2240 microscopic images representing 112 distinct tree species from 85 genera and 30 families, divided into two groups: hardwood and softwood. These images were captured using an Olympus CX40 microscope with a 100× magnification. The resulting color images are saved in uncompressed Portable Network Graphics (PNG) format with a resolution of 1024 × 768 pixels.

### 2.2. Dataset Augmentation Based on Basic Image Processing Methods

Basic image processing methods are simple yet effective techniques for augmenting datasets, especially when the dataset is small or difficult to collect [31]. These methods include image rotation, flipping, contrast adjustment, and brightness modification. The newly generated images are similar to the original ones but are not identical [32]. Due to the limited number of images in the dataset—only 20 images—directly inputting them into a GAN model would result in poor robustness. Therefore, the original dataset was first augmented using basic image processing methods before being fed into the GAN model for training. The basic image processing techniques used in this study for dataset augmentation include image flipping, translation, brightness adjustment, chroma adjustment, contrast adjustment, sharpness adjustment, and the addition of Gaussian noise.

We acknowledge that the dataset used for training consists of only 20 or 280 base microscopic wood images, which places all GANs in a highly constrained regime. This setting reflects realistic conditions in microscopic wood anatomy, where many species have extremely limited imaging samples. As a result, the generated samples and quantitative metrics (e.g., IS, KID) may exhibit instability. Our goal is therefore not to achieve maximal fidelity, but to study the robustness and behavior of different GAN architectures under this extreme low-data scenario.

### 2.3. Dataset Augmentation Based on Generative Adversarial Networks

**Figure 2:**
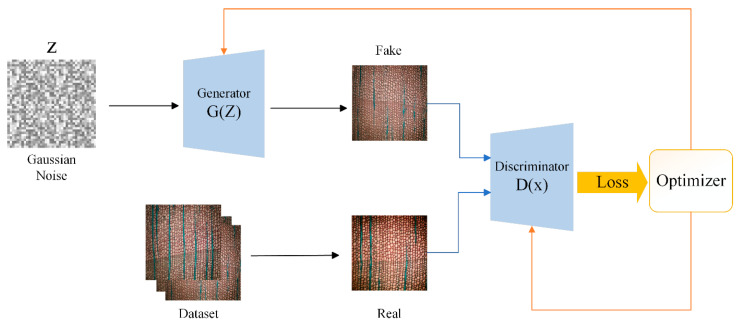
The main principle of GAN.

The architecture of a GAN consists of two key components: the generator and the discriminator. The generator learns to map random noise into realistic data, while the discriminator attempts to accurately distinguish between real data and fake data generated by the generator. During training, these two components engage in a competitive process, with the generator striving to deceive the discriminator while the discriminator works to improve its ability to distinguish real from fake data. This adversarial training process drives the generator to produce fake data that increasingly resembles real data in terms of its features. Figure 2 shows the schematic diagram of the GAN architecture.

The GAN loss function $V\left(G,D\right)$ is defined as shown in Equation (1):

(1) $$\underset{G}{min}\;\underset{D}{max}\;V(G,D)=E_{x∼P_{data}(x)}[logD(x)]+E_{Z∼P_{z}(z)}[log(1-D\left(G(z)\right))]$$

where min represents the minimization of V; max represents the maximization of V; G is the generator; D is the discriminator; x is the real data, with $P_{data}(x)$ denoting its distribution; and z is the noise input to G, with $P_{z}(z)$ denoting its distribution.

In this study, the dataset augmented using basic image processing methods was used to train five models—BGAN, DCGAN, WGAN-GP, LSGAN, and StyleGAN2—for 4000 epochs, with the target generated image size set to 256 × 256. During the training process, the model’s weight parameters were saved so that the trained model could be used to generate images after training. After training, the performance of the generated images will be evaluated from the perspectives of image quality as well as KID [33], IS [34], and SSIM [35]. The goal was to conduct a comparative analysis of the performance of each model in order to select the best model for dataset augmentation. The models were built using the PyTorch 1.12 framework. The experimental environment and training hyperparameters are summarized in Table 2.

All models were trained for 4000 epochs. Although this may seem extensive for a small dataset, preliminary trials showed that shorter training (e.g., 500–1500 epochs) did not yield stable convergence or recognizable texture structures. Longer training was necessary for models such as WGAN-GP and StyleGAN2 to reach stable discriminator–generator equilibrium. Overfitting was monitored through visual inspection of intermediate results and avoidance of mode collapse, rather than through a large validation set.

#### 2.3.1. BGAN

BGAN is an improved GAN designed to address the challenges of training instability and mode collapse in traditional GANs. While BGAN was originally proposed with capabilities for handling discrete data, its core contribution—the boundary-seeking objective function—provides significant benefits for continuous image data as well. In BGAN, the generator’s objective function is adjusted to compute the target based on the discriminator’s evaluation. The generator attempts to produce samples near the boundary of the target distribution, which resemble real samples but are not identical, thereby increasing the difficulty for the discriminator to distinguish them. This approach prevents the generator from producing samples that are too close to the center of the target distribution, which would be too similar to real data, thus helping to avoid mode collapse. To improve training stability, BGAN employs methods such as policy gradient. Policy gradient is a reinforcement learning algorithm used to optimize the generator’s objective function. By using policy gradient, we can more effectively guide the generator to produce samples closer to the boundary of the target distribution, thus avoiding instability and mode collapse during training.

#### 2.3.2. DCGAN

DCGAN builds the generator and discriminator using convolutional neural networks, significantly improving the quality of generated images. DCGAN employs convolutional and deconvolutional layers to process image data and incorporates techniques like batch normalization and LeakyReLU activation functions to address the instability issues encountered in GAN training. It is capable of generating more realistic and detail-rich images and is widely used in image generation, image editing, and image super-resolution tasks.

#### 2.3.3. WGAN-GP

WGAN-GP is an enhanced version of the GAN aimed at improving training stability and the quality of generated images. Unlike traditional GANs, WGAN-GP introduces Wasserstein distance and gradient penalty to solve mode collapse and training instability. In WGAN-GP, the adversarial learning objective between the discriminator and the generator is to minimize the Wasserstein distance rather than the Jensen-Shannon divergence used in traditional GANs. This distance measure allows for a more accurate comparison of the difference between the distribution of the generated images and the real data distribution. WGAN-GP incorporates a gradient penalty term in the loss function, which helps keep the discriminator’s gradient within a specific range, ensuring training stability and gradient smoothness. By optimizing the Wasserstein distance and introducing gradient penalty, WGAN-GP effectively solves the training instability problems of traditional GANs, improving both the quality and stability of the generated images and making the training process more reliable and efficient.

#### 2.3.4. LSGAN

LSGAN is a variant of GAN that replaces the traditional binary cross-entropy loss with a least squares loss function. By minimizing the mean squared error between the generated images and real images, LSGAN enables the generator to learn the distribution of real images more stably, resulting in clearer and more realistic images. Since the least squares loss function provides a smoother optimization target, LSGAN is less prone to mode collapse and gradient vanishing issues during training.

#### 2.3.5. StyleGAN2

StyleGAN2 is an advanced generative adversarial network model developed by NVIDIA, known for its ability to generate high-quality, high-resolution facial images. StyleGAN2 redesigns the Adaptive Instance Normalization (AdaIN) layer and introduces weight modulation technology to solve the “water-drop” artifact problem, making the images more natural and reducing artifacts and noise. The network architecture of StyleGAN2 is also optimized, consisting of three main components: the mapping network, the style generation network, and the synthesis network. The mapping network is responsible for mapping the input random noise vector into an intermediate latent space (W space), which is more decoupled than the original Z space and helps generate more natural and diverse images. The style generation network retrieves style vectors from the W space and injects style information into each layer of the generator using AdaIN and weight modulation technology, thus controlling the image generation process. Finally, the synthesis network generates images from low resolution to high resolution based on the style information provided by the style generation network. StyleGAN2 is a milestone model in the field of image generation, and with its exceptional image generation quality and detail expression, it has become the mainstream method for high-resolution image synthesis and facial generation.

### 2.4. Quality Assessment Indicators

The quality assessment of generated wood anatomy images was conducted using established quantitative metrics, with all comparisons based on the original collection of wood anatomy [20] images as the evaluation benchmark.

#### 2.4.1. KID

To provide domain-specific evaluation of generated image quality, we computed the KID using feature embeddings extracted from a DenseNet classifier pre-trained on the UFPR wood species dataset. This domain-adapted approach ensures that the evaluation metric is sensitive to wood anatomical characteristics rather than generic image features. The DenseNet model was trained to classify 37 wood species from the same UFPR dataset, thus providing highly relevant feature representations for wood microscopic image assessment.

Unlike Fréchet Inception Distance (FID), which assumes Gaussian distributions, KID uses the maximum mean discrepancy (MMD) with a polynomial kernel, providing an unbiased estimator that is more robust for smaller sample sizes. The KID is defined as:

(2) $$KID\left(P_{r},\;P_{g}\right)=E\left[k\left(x,x^{\prime }\right)+\;k\left(y,y^{\prime }\right)-\;2k\left(x,y\right)\right]$$

where $P_{r}$ represents the real data distribution, $P_{g}$ represents the generated data distribution, and $x,x^{\prime }\sim P_{r}$ and $y,y^{\prime }\sim \;P_{g}$ are independently sampled pairs. The function $k\left(x,y\right)$ is the polynomial kernel defined as:

(3) $$k\left(x,y\right)=\;{\left(\frac{1}{d}xᵀy\;+\;1\right)}^{3}$$

where d is the feature dimension and xᵀy denotes the inner product of vectors x and y.

In practice, we use the unbiased estimator:

(4) $$\hat{KID}(X,Y)=\frac{1}{n(n-1)}\sum_{i\neq j}\;k(x_{i},x_{j})+\frac{1}{m(m-1)}\sum_{i\neq j}\;k(y_{i},y_{j})-\frac{2}{nm}\sum_{i,j}\;k(x_{i},y_{j})$$

where $X=\{x_{1},\ldots ,x_{n}\}$ and $Y=\{y_{1},\ldots ,y_{m}\}$ are feature embeddings extracted from n real and m generated images, respectively, using our domain-specific DenseNet model.

#### 2.4.2. IS

IS evaluates the quality of generated images from two aspects:

Realism: The generated image x is input into an Inception classification network, which outputs a 1000-dimensional vector y, where each dimension corresponds to the probability of the image belonging to a particular class. For a real image, the probability of belonging to a particular class should be very high, and the probability for other classes should be very low. In other words, the entropy of $p\left(y|x\right)$ should be small.

Diversity: If a model generates sufficiently diverse images, the distribution of the generated images across different categories should be uniform. This means that the entropy of the marginal distribution p(y) of the probabilities across all categories should be large.

The IS is the average Kullback–Leibler (KL) divergence between these two distributions, as described by the following formula:

(5) $$IS\left(G\right)=\exp\left(E_{x\sim p_{g}}D_{KL}\left(p\left(y|x\right)||p(y)\right)\right)$$

where $E_{x\sim p_{g}}$ is the expectation of the generated image distribution, and $D_{KL}$ is the Kullback–Leibler divergence that measures the difference between the conditional probability distribution $p\left(y|x\right)$ and the marginal distribution p(y).

#### 2.4.3. SSIM

The SSIM is a quality metric used to measure the similarity between two images. It is considered to be related to the quality perception of the human visual system (HVS). The design of SSIM models any image distortion as a combination of three factors: correlation loss, luminance distortion, and contrast distortion. The definition of SSIM is as follows:

(6) $$SSIM\left(f,g\right)=l(f,g)c(f,g)s(f,g)$$

(7) $$l\left(f,g\right)=\frac{2\mu_{f}+\mu_{g}+C_{1}}{\mu_{f}^{2}+\mu_{g}^{2}+C_{1}}$$

(8) $$c\left(f,g\right)=\frac{2\sigma_{f}\sigma_{g}+C_{2}}{\sigma_{f}^{2}+\sigma_{g}^{2}+C_{2}}$$

(9) $$s\left(f,g\right)=\frac{\sigma_{fg}+C_{3}}{\sigma_{f}\sigma_{g}+C_{2}}$$

Formulas (5)–(7) represent the luminance comparison function, which measures the similarity of the average luminance between two images ($\mu_{f}$ and $\mu_{g}$); the contrast comparison function, which calculates the similarity of the contrast between two images based on their standard deviations ($\sigma_{f}$ and $\sigma_{g}$); and the structural comparison function, which measures the correlation coefficient between the two images f and g. The $\sigma_{fg}$ parameter is the covariance between f and g. A value of 0 indicates no correlation between the images, and 1 indicates that f=g.

Each generated image was paired with one of the original 20 reference images representing the same species and anatomical characteristics.

## 3. Results and Discussion

### 3.1. Data Augmentation Using Basic Image Processing Methods

**Figure 3:**
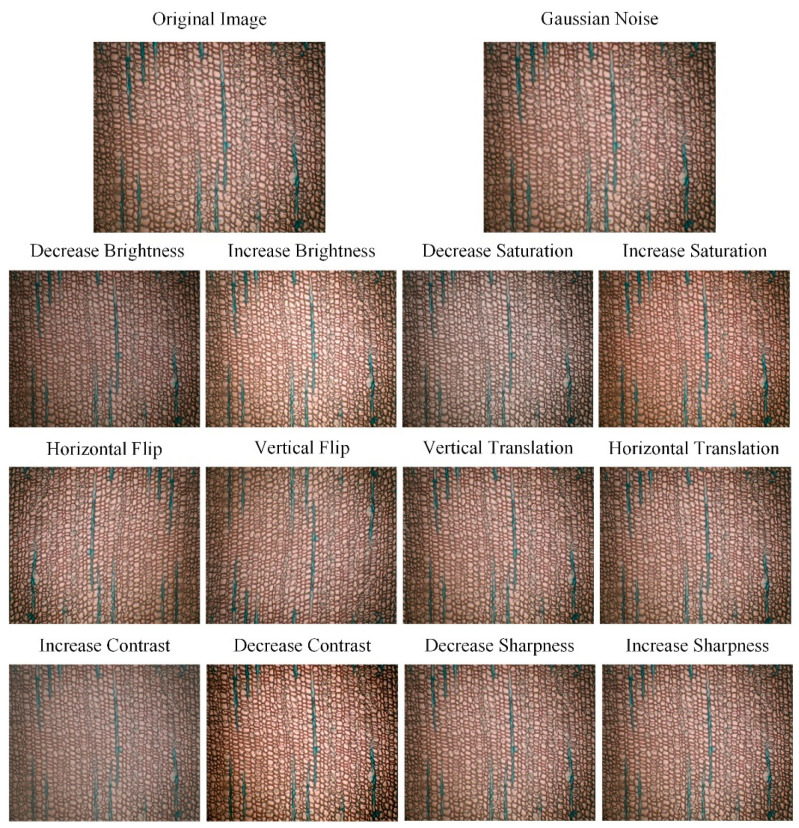
Augmentation of Microscopic Images of Ginkgo Wood Cells Using Basic Image Processing Methods. The original image (top-left) is augmented using various transformations: Gaussian noise, brightness adjustment (increase and decrease), saturation adjustment (increase and decrease), flipping (horizontal and vertical), translation (horizontal and vertical), contrast adjustment (increase and decrease), and sharpness adjustment (increase and decrease).

Basic image processing methods were used to augment the original 20 images of ginkgo wood cellular micrographs. The processing included flipping the images vertically and horizontally, shifting them horizontally and vertically, reducing or enhancing the brightness, chroma, contrast, and sharpness, as well as adding Gaussian noise. By applying these transformations, one original image could generate 13 new images. The dataset was expanded from 20 images to 280 images with the original images included. The processed images are shown in Figure 3. The figure presents the original images after being processed with various image manipulation techniques. The top-left image shows the original image, and the remaining images correspond to the processing methods described in the text above.

### 3.2. Ablation Study on Data Augmentation Strategies

To systematically evaluate the contribution of different augmentation techniques, we conducted an ablation study using WGAN-GP across four progressively augmented datasets: the original configuration with 20 images and no augmentation served as the baseline; the basic augmentation with 60 images incorporated geometric transformations including flipping; the medium augmentation with 140 images expanded upon the basic set by adding translation and brightness adjustments; and the complete augmentation with 280 images encompassed all 13 augmentation methods employed in this study. The quantitative results of this progressive augmentation analysis are presented in Table 3.

**Figure 4:**
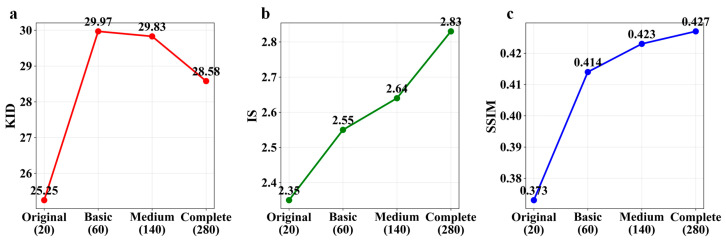
Performance evaluation of WGAN-GP across different augmentation levels: (**a**) KID, (**b**) IS, and (**c**) SSIM.

The performance trends across different augmentation levels are visualized in Figure 4, which presents the KID, IS, and SSIM metrics for each configuration.

Analysis of the results reveals several important patterns. First, the IS and SSIM metrics demonstrate consistent improvement with increasing augmentation complexity, indicating enhanced image quality and structural similarity. The IS improved by 20.4% from the original to complete augmentation configuration (from 2.35 to 2.83), while SSIM showed a 14.5% improvement (from 0.373 to 0.427).

However, the KID metric exhibits a non-monotonic behavior, initially deteriorating from 25.25 to 29.97 during the basic augmentation stage, remaining high at 29.83 with medium augmentation, and then partially recovering to 28.58 with complete augmentation. This phenomenon may be attributed to the increased distribution complexity introduced by partial augmentation strategies, which temporarily widens the distribution gap between generated and real images in the domain-specific feature space. The complete augmentation configuration achieves the optimal balance, yielding the best IS and SSIM scores while reducing the KID value compared to intermediate stages, though it does not fully return to the original baseline.

These findings underscore the importance of comprehensive augmentation strategies and provide valuable insights for optimizing data preparation in wood microscopic image analysis, highlighting that while intermediate enhancements may temporarily affect distribution similarity, full augmentation ultimately leads to the best overall performance in terms of image quality and structural fidelity.

### 3.3. Data Augmentation Based on GAN

The augmented dataset containing 280 microscopic images was used to train five GAN models—BGAN, DCGAN, WGAN-GP, LSGAN, and StyleGAN2—under identical training conditions. Instead of presenting image-by-image descriptions, this section provides an integrated analysis focusing on (1) overall training dynamics, (2) model-specific performance characteristics, and (3) comparative evaluation based on fidelity, diversity, and structural realism.

#### 3.3.1. Overall Training Dynamics

**Figure 5:**
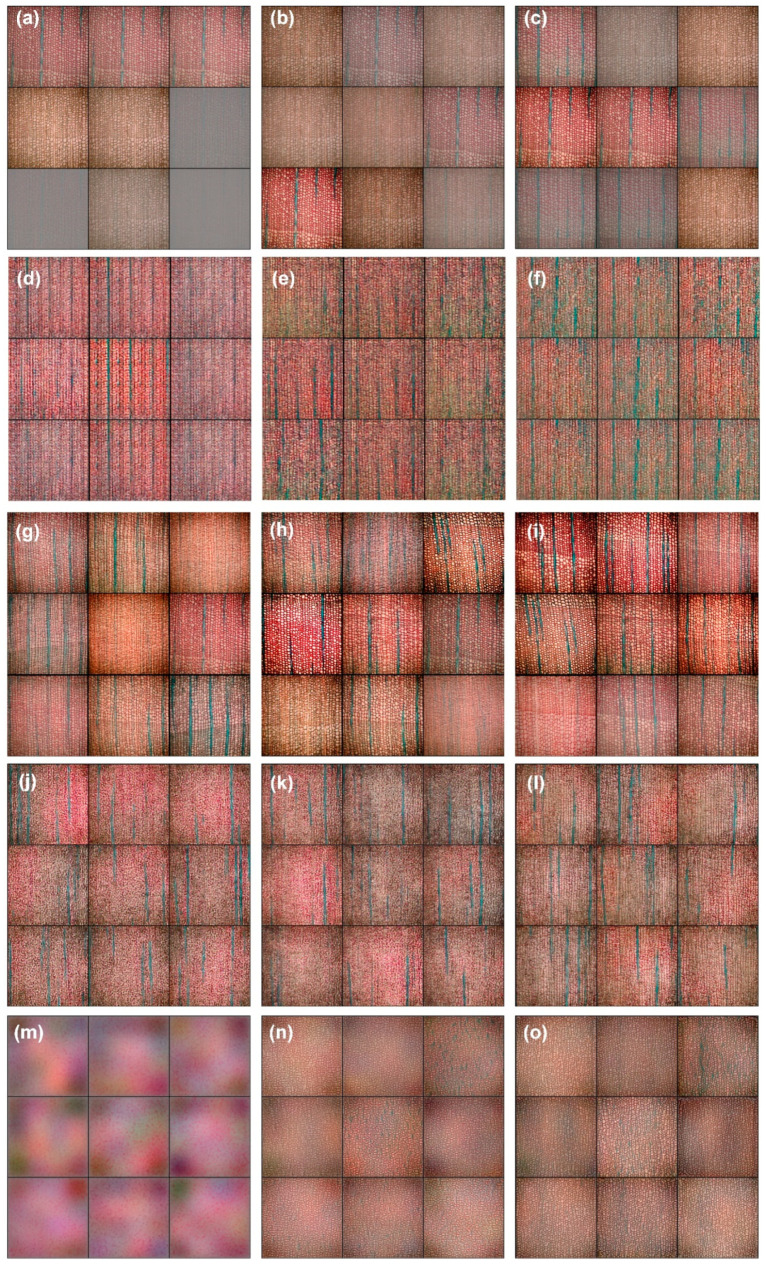
Generated Microscopic Images of Ginkgo Wood Cells by Different GAN Models After 1000, 2000, and 3000 Training Epochs. The figure illustrates the generated images of ginkgo wood cells using five GAN models: BGAN (**a**–**c**), DCGAN (**d**–**f**), WGAN-GP (**g**–**i**), LSGAN (**j**–**l**), and StyleGAN2 (**m**–**o**) at different training stages (1000, 2000, and 3000 epochs).

Across all models, the quality of generated images gradually improved with longer training. Although the visual evolution at 1000, 2000, and 3000 epochs is illustrated in Figure 5, the general trends can be summarized as follows:

Early stage (~1000 epochs): All models produced noisy and unstable textures with incomplete cellular structures, as expected in low-data settings.

Middle stage (~2000 epochs): Texture clarity improved, noise decreased, and basic anatomical structures (e.g., tracheids, rays) became recognizable. However, diversity remained highly architecture-dependent.

Late stage (~3000 epochs): WGAN-GP achieved stable refinement with consistently realistic structures. BGAN improved moderately but showed repeated patterns. DCGAN, LSGAN, and StyleGAN2 exhibited stagnation or partial mode collapse.

To avoid redundancy, detailed epoch-by-epoch commentary is omitted. Instead, the following subsection provides a model-level analysis of structural quality and generative behavior.

#### 3.3.2. Model-Specific Performance Analysis

**Figure 6:**
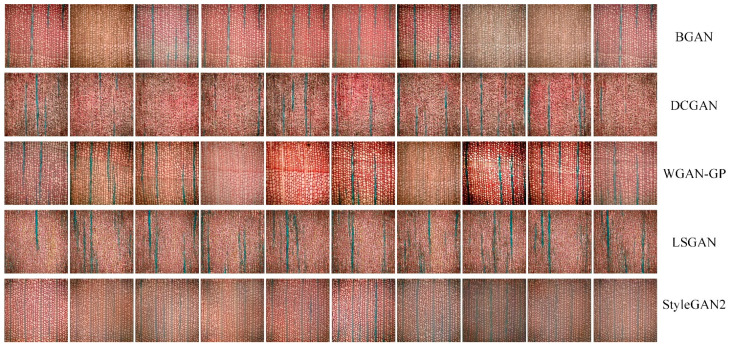
Generated Images from Five GAN Models (BGAN, DCGAN, WGAN-GP, LSGAN, and StyleGAN2). This figure illustrates the generated images of wood microscopic structures using five GAN models: BGAN, DCGAN, WGAN-GP, LSGAN, and StyleGAN2. Each row represents the output of a specific model, showcasing the differences in image quality, texture consistency, and structural detail restoration among the models.

Figure 6 shows the images generated by five GAN models: BGAN, DCGAN, WGAN-GP, LSGAN, and StyleGAN2.

BGAN produces structurally reasonable cell textures with gradually improving clarity over the training period. However, the model tends to concentrate on limited texture modes, resulting in low diversity and partial mode collapse. This behavior is consistent with JS-divergence–based training, which is sensitive to small datasets and encourages conservative solutions with limited variation.

DCGAN struggles to capture anatomical variability. Although basic textures emerge, generated images remain blurry with weak cell delineation. Strong mode collapse is observed, and earlywood–latewood transitions are rarely reproduced. This aligns with the known instability of DCGAN on limited datasets due to discriminator overpowering.

WGAN-GP achieves the clearest and most realistic cell structures among all models. The Wasserstein distance ensures smooth gradients, while the gradient penalty prevents discriminator explosion—critical advantages when training with only 280 base images. As a result, WGAN-GP maintains both fidelity and diversity, avoids mode collapse, and naturally reconstructs earlywood–latewood transitions and ray alignment.

LSGAN shows improved stability over DCGAN but still fails to reproduce natural transitions between earlywood and latewood. Texture continuity is weak, and diversity remains limited. Its least-squares objective reduces vanishing gradients but is still insufficient for such an extremely small dataset.

StyleGAN2 generates textures with moderate coherence but suffers from slow convergence and a tendency to collapse into repetitive patterns. This performance is a direct consequence of training a highly complex, data-hungry architecture on a very small dataset. The model’s sophisticated components, such as its style-based generator, are inherently data-hungry and become underconstrained with only 280 samples, inevitably leading to overfitting and partial mode collapse. Although strategies like pretraining on larger datasets could alleviate this, they are not feasible within the constraints of our single-species investigation and are therefore reserved for future research.

In conclusion, WGAN-GP consistently achieves the best balance of fidelity and diversity among the evaluated models. This can be explained by its use of the Wasserstein distance, which provides smoother and more informative gradients than JS-based losses used in BGAN or DCGAN. The gradient penalty further regularizes the discriminator, preventing it from becoming overly strong—a common failure mode when training with extremely small datasets. These properties make WGAN-GP particularly suitable for low-data microscopic images, where maintaining stable updates is essential for avoiding collapse. WGAN-GP excels in generating wood micrograph images, outperforming the other models in clarity, detail restoration, and diversity. Its generated images are closest to real images, demonstrating its exceptional generation capabilities.

Table 4 shows the training time for different models. BGAN has the shortest training time and relatively fast training speed, producing models with moderate quality. WGAN-GP, on the other hand, has a moderate training time but achieves the best generation performance, demonstrating strong detail restoration and diversity capabilities. In comparison, StyleGAN2 has the longest training time, more than 14 times longer than BGAN. This is mainly due to the more complex structure and a larger number of parameters in the StyleGAN2 model, leading to a significant increase in training time.

**Figure 7:**
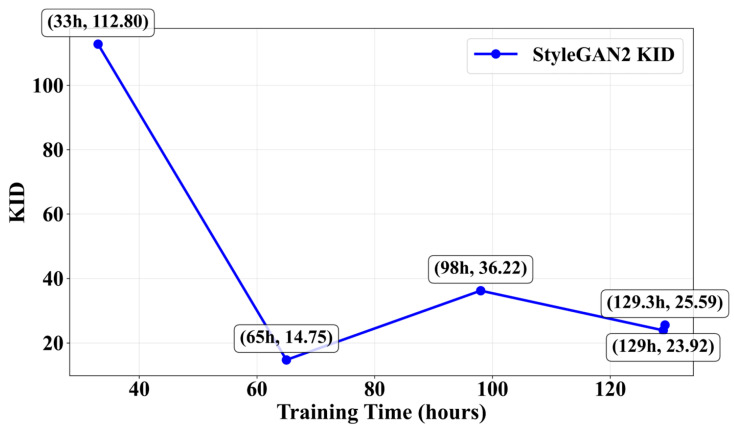
StyleGAN2 training efficiency pareto curve.

To further analyze the training efficiency of StyleGAN2, we conducted a Pareto analysis of its training duration versus image quality, as shown in Figure 7. The curve illustrates the relationship between training time and the corresponding KID score, providing insights into how image quality evolves during the training process. The analysis reveals that the KID score does not monotonically decrease with extended training, suggesting that there may be an optimal training duration beyond which additional training yields diminishing returns. This observation aligns with the known characteristics of GAN training, where prolonged training can sometimes lead to overfitting or mode collapse.

While this analysis provides initial insights into the duration–quality relationship of StyleGAN2, we acknowledge that KID is only one of several evaluation metrics, and a more comprehensive analysis incorporating multiple quality measures would be beneficial for future work. Nevertheless, this Pareto curve offers valuable guidance for researchers seeking to balance computational cost and model performance when training complex generative models.

The high computational cost and long training time of complex models make them less cost-effective for small-scale data tasks. However, understanding the duration–quality trade-off through such Pareto analysis allows researchers to make informed decisions about training strategies and resource allocation.

#### 3.3.3. Qualitative Evaluation

To comprehensively evaluate the performance of the five GAN models, we generated 1000 images using each model and employed a multifaceted assessment framework comprising six quantitative metrics: KID, IS, SSIM, Density, Coverage, and LPIPS diversity. The comparative results presented in Table 5 reveal distinct performance profiles across different models, highlighting the complex trade-offs in generative modeling for wood anatomy imagery.

The KID and Density–Coverage metrics provide complementary perspectives on distribution matching. BGAN achieved the most favorable KID score (20.42), indicating superior alignment with the real image distribution. This is further supported by its strong Density (0.52) and Coverage (0.90) values, suggesting that BGAN generates samples that not only match the real distribution well but also comprehensively cover the underlying data manifold.

WGAN-GP demonstrated an interesting performance profile, achieving the highest Coverage (0.95) despite a moderate KID score (28.58). This indicates that while WGAN-GP’s generated distribution may not perfectly align with the real data, it excels at exploring the entire data manifold. The model also achieved the highest IS (3.04), reinforcing its capability to produce diverse and recognizable wood anatomy patterns.

The LPIPS diversity metric reveals important insights into the perceptual characteristics of generated images. BGAN and StyleGAN2 exhibited the lowest LPIPS diversity values (0.32 and 0.33, respectively), indicating more consistent but potentially less varied output. In contrast, WGAN-GP and DCGAN showed higher diversity scores (0.49 and 0.50), though DCGAN’s performance must be interpreted cautiously given its near-zero Density and Coverage values.

The consistently low SSIM values across all models (0.36–0.39) with minimal differentiation suggest that pixel-level structural similarity may not be the most discriminative metric for wood anatomy image generation. This could be attributed to the complex, stochastic nature of wood anatomical patterns, where exact pixel-level correspondence with reference images may not adequately capture perceptual quality.

The absolute values of these metrics require careful interpretation within the specialized domain of wood anatomy imaging. The ISs (1.33–3.04) are substantially lower than those typically reported for natural images, reflecting both the complexity of wood anatomical features and the challenges of limited training data. Similarly, the Density and Coverage metrics should be considered relative to each other rather than as absolute measures of quality.

Our multi-faceted evaluation reveals that model selection should be guided by application requirements. BGAN emerges as the preferred choice for applications requiring high fidelity to real wood anatomy distributions, while WGAN-GP offers advantages for scenarios prioritizing diversity and manifold coverage. The relatively poor performance of DCGAN across all metrics confirms its limitations for this specialized domain, while StyleGAN2’s complex architecture appears suboptimal for small-scale image generation tasks.

These quantitative findings underscore the importance of considering multiple complementary metrics when evaluating generative models, particularly for specialized domains where no single metric fully captures the nuances of image quality and diversity.

#### 3.3.4. Metric Behavior and Limitations

The quantitative results show that BGAN obtains the lowest KID while WGAN-GP achieves the highest IS. This apparent inconsistency arises because KID and IS capture different aspects of generation quality. In extremely small datasets, KID tends to favor generators that produce compact, low-diversity samples close to the mean of the real distribution, whereas IS rewards perceptual diversity across generated samples. Therefore, BGAN’s low-variance outputs yield a lower KID, while WGAN-GP’s broader structural variation leads to a higher IS. This divergence is expected under limited-data conditions.

The absolute IS values (<3) are low compared to typical natural-image benchmarks because microscopic wood images contain repetitive and low-entropy textures. As a result, the IS of real images themselves is low, making the generated-image IS inherently dataset-dependent rather than universally comparable. SSIM values in the 0.36–0.39 range also reflect the limited dynamic range of SSIM for fine-grained textures, where small structural differences are compressed. Thus, these metrics should be interpreted comparatively rather than absolutely.

To ensure robustness of interpretation, we cross-validated the quantitative findings using LPIPS diversity, Density–Coverage, and visual anatomical criteria commonly employed in microscopic wood analysis. These complementary evaluations consistently support the conclusion that WGAN-GP provides the best balance of fidelity and diversity in the limited-data regime.

#### 3.3.5. Impact of Different Dataset Sizes on Model Training

**Figure 8:**
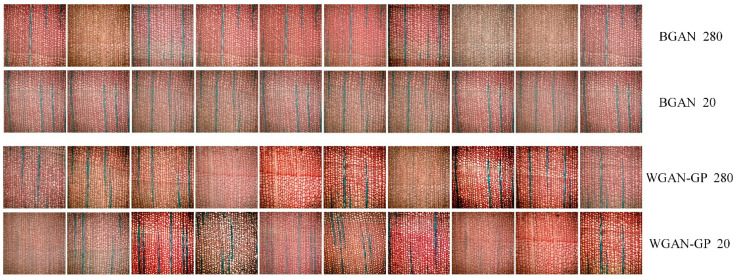
Generated Images from BGAN and WGAN-GP with Different Dataset Sizes (20 and 280 Images). This figure compares the generated images of wood microscopic structures using BGAN and WGAN-GP models. Rows labeled “280” represent models trained on the expanded dataset of 280 images derived from the original 20 images, while rows labeled “20” represent models trained on the original dataset of 20 images.

When using a dataset of 280 images, both BGAN and WGAN-GP performed relatively well. Therefore, this section trains both models using the original dataset of 20 images. Figure 8 shows the images generated by the BGAN and WGAN-GP models trained on datasets of different sizes. By expanding the dataset to 280 images, both BGAN and WGAN-GP generated images that were significantly clearer and more detailed than when using only the original 20 images. The images generated after data augmentation appeared more natural, with richer details, and were able to better show the early and late wood cells. The arrangement of the wood rays conformed to the characteristics of the species, and the occurrence of mode collapse was greatly reduced.

When trained on only the original 20-image dataset, the images generated by BGAN were slightly blurry with insufficient detail. While WGAN-GP generated relatively clear images, it still exhibited issues like texture loss and lack of consistency. However, when the dataset was expanded to 280 images, the quality of the images generated by BGAN significantly improved, and WGAN-GP was able to achieve both high quality and diversity. This indicates that the original 20 images were insufficient to support the GAN models in learning the complex structural features of wood cells, leading to blurry and inconsistent generated images. Data augmentation significantly improved the model’s ability to learn the microstructural features of wood, making the generated images much closer to the real ones.

## 4. Conclusions

In this study, we systematically addressed the challenge of extreme data scarcity in wood microscopic image analysis by establishing a comprehensive benchmark for evaluating GAN models under low-data conditions. Through a cross-architecture comparison of five representative models—BGAN, DCGAN, WGAN-GP, LSGAN, and StyleGAN2—we found that WGAN-GP provides the most favorable balance among image clarity, structural diversity, and anatomical fidelity, effectively reconstructing key features such as earlywood, latewood, and wood rays while exhibiting strong resilience to mode collapse. BGAN demonstrated reliable performance in scenarios where texture consistency is prioritized, whereas DCGAN, LSGAN, and the more complex StyleGAN2 suffered from instability, overfitting, and repetitive patterns. These observations underscore that models developed for natural-image domains do not readily transfer to highly structured microscopy data under severe sample constraints.

Beyond ranking model performance, this study establishes a domain-aware evaluation paradigm incorporating wood-specific feature embeddings into KID and provides anatomy-grounded analyses of model failure modes. Together, these components offer a practical and interpretable reference for selecting GAN architectures suitable for wood microscopic imagery under limited data.

While the benchmark yields actionable insights, certain boundaries should be acknowledged.

First, the present work focuses on generative fidelity and does not extend to evaluating the effect of synthetic data on downstream classification tasks. Such an assessment, although beyond the current scope, represents an important direction for establishing the functional utility of GAN-generated samples.

Second, the findings are based on a single-species, small-scale dataset, and therefore, the generality of the conclusions should be interpreted within this constrained context. Extending the analysis to multiple taxa or other biologically structured materials will be essential for validating the broader applicability of these results.

Building upon the foundation established here, future work may explore wood-structure-sensitive loss formulations, hybrid augmentation pipelines that combine generative and traditional methods, and multi-domain evaluations to examine transferability across diverse datasets. As such, this benchmark not only offers immediate guidance for methodological choices in wood microscopy but also outlines a clear trajectory for advancing GAN-based augmentation in data-limited scientific imaging.

## Figures and Tables

**Table 1:** Overview of GAN architectures considered in this work, detailing their fundamental contributions and documented limitations.

Model	Core Contributions	Main Limitations & Criticisms
BGAN [26]	Proposed a boundary-seeking objective function, particularly suitable for discrete data. It generates samples by having the generator approximate the “boundary” of the real data distribution, aiding training stability and mitigating mode collapse.	Performance Dependency: Its performance heavily relies on the effective use of RL techniques like policy gradient, increasing implementation complexity.
DCGAN [27]	Established the foundation for CNNs in GAN architectures, proposing a set of architectural guidelines (e.g., using strided convs, batch norm, ReLU/LeakyReLU), which laid the groundwork for subsequent CNN-based GAN research.	Training Instability: Training can still be unstable and suffer from mode collapse despite improvements. Resolution Limitation: The original architecture struggles to generate high-resolution, highly realistic images.
WGAN-GP [28]	Introduced the Wasserstein distance as the loss function, providing theoretically smoother gradients and significantly improving training stability. Proposed Gradient Penalty to enforce the Lipschitz constraint, avoiding the optimization issues caused by weight clipping.	Computational Overhead: The gradient penalty term adds extra cost as it requires computing gradient norms on interpolated points between real and fake data.
LSGAN [29]	Replaced the traditional cross-entropy loss with a least squares loss. This provides the discriminator with smoother and non-saturating gradients, helping to stabilize training and generate higher-quality images.	Mode Collapse: While alleviated, LSGAN can still suffer from mode collapse, especially on complex datasets.
StyleGAN2 [30]	Redesigned the generator architecture to eliminate “water droplet” artifacts present in StyleGAN1. Introduced weight demodulation and path length regularization, enabling more precise control over styles and generating exceptionally high-quality and diverse images.	Extreme Complexity: The model is very complex and requires substantial computational resources and time to train. Data Hungry: Heavily relies on large-scale, high-quality datasets to reach its potential; prone to overfitting or underperformance on small datasets. Latent Space Interpretability: While offering control, its advanced latent space structure remains complex and is not fully interpretable.

**Table 2:** Experimental Environment and Training Configuration.

Parameter	Value	Description
GPU	NVIDIA GeForce RTX 4060Ti 8 GB	Graphics processing unit for accelerated model training
CPU	13th Gen Intel(R) Core (TM) i5-13490F 2.50 GHz	Central processing unit for data preprocessing operations
Python	3.9	Programming language environment for implementation
PyTorch	1.12	Deep learning framework for neural network operations
Total Epochs	4000	Maximum training epochs
Batch Size	64	Samples per batch
Learning Rate	0.0002	Adam optimizer learning rate
Optimizer	Adam	β_1_ = 0.5, β_2_ = 0.999
Image Size	256 × 256	Output image resolution
Gradient Penalty	λ = 10	WGAN-GP gradient penalty coefficient

**Table 3:** Progressive Impact of Data Augmentation on WGAN-GP Performance Across Dataset Scales. Note: ↓ (KID) = smaller better; ↑ (IS, SSIM) = larger better.

Augmentation Configuration	Number of Images	KID (↓)	IS (↑)	SSIM (↑)
Original Only	20	25.25	2.35	0.373
Flips	60	29.97	2.55	0.414
Flips + Translation + Brightness	140	29.83	2.64	0.423
All 13 Methods	280	28.58	2.83	0.427

**Table 4:** Training Time of Different GAN Models.

Models	Training Time (s)
BGAN	32,187
DCGAN	152,289
WGAN-GP	37,382
LSGAN	72,079
StyleGAN2	465,796

**Table 5:** Comparison of KID, IS, SSIM, Density, Coverage and LPIPS for Generated Images.

Models	KID	IS	SSIM	Density	Coverage	LPIPS
BGAN	20.42	1.74	0.39	0.52	0.90	0.32
DCGAN	45.97	1.67	0.37	0	0	0.50
WGAN-GP	28.58	3.04	0.39	0.26	0.95	0.49
LSGAN	30.51	1.83	0.37	0.03	0.25	0.46
StyleGAN2	25.59	1.33	0.36	0.44	0.50	0.33

## Data Availability

The original data presented in the study are openly available in Forest Species Microscopic Image Database maintained by the Federal University of Paraná (UFPR), Brazil at https://web.inf.ufpr.br/vri/databases/forest-species-database-microscopic/ (accessed on 10 September 2023). The dataset and its structure are described in detail in the original publication by Martins et al. [12].
